# Minimally Invasive Bimanual Fetal Surgery—A Review

**DOI:** 10.3390/children9091377

**Published:** 2022-09-13

**Authors:** Susanne Eva Brunner, Lidya-Olgu Durmaz, Andreas Meinzer, Milena Arp, Thomas Franz Krebs, Robert Bergholz

**Affiliations:** 1 Department of General, Visceral, Thoracic, Transplant and Pediatric Surgery, University Medical Center Schleswig-Holstein (UKSH), Kiel Campus, Arnold-Heller-Strasse 3, 24105 Kiel, Germany; 2 Department of Pediatric Surgery, Children’s Hospital of Eastern Switzerland, Claudiusstrasse 6, 9006 St. Gallen, Switzerland

**Keywords:** pediatric surgery, minimally invasive surgery, endoscopic surgery, open surgery, prenatal surgery, fetoscopy surgery, surgical techniques, bimanual surgery

## Abstract

Background: The aim of this review is to discuss experimental and clinical techniques and interventions of fetal surgery which have been performed minimally invasively by the means of a three-port approach for the fetoscope and instruments for the left and right hand of the surgeon (bimanual minimally invasive fetal surgery). Methods: a print and electronic literature search was performed; the titles and abstracts were screened and included reports were reviewed in a two-step approach. First, reports other than minimally invasive fetal surgery were excluded, then a full text review and analysis of the reported data was performed. Results: 17 reports were included. The heterogeneity of the included reports was high. Although reports on human fetoscopic surgical procedures can be found, most of them do not pick out bimanual fetal surgery as a central theme but rather address interventions applying a fetoscope with a working channel for a laser fiber, needle or flexible instrument. Most reports were on experimentation in animal models, the human application of minimally invasive fetoscopic bimanual surgery is rare and has at best been explored for the prenatal treatment of spina bifida. Some reported bimanual fetoscopic procedures were performed on the exteriorized uterus via a maternal laparotomy and can therefore not be classified as being truly minimally invasive. Discussion: our results demonstrate that minimally invasive fetoscopic bimanual surgery is rare, even in animal models, excluding many other techniques and procedures that are loosely termed ‘minimally invasive fetal surgery’ which we suggest to better label as ‘interventions’. Thus, more research on percutaneous minimally invasive bimanual fetoscopic surgery is warranted, with the aim to reduce the maternal, uterine and fetal trauma for correction of congenital malformations.

## 1. Introduction

Fetal surgery is synonymously used for prenatal interventions of various techniques such as ultrasound-guided percutaneous needle insertion and laser ablation of placental vessels as well as maternal laparotomy and hysterotomy for the open surgical repair of myelomeningocele [1,2,3,4].

Whether a prenatal condition has been treated by minimally invasive procedures that have been performed utilizing instruments using the left and right hand of the surgeon, with an additional port for the camera or fetoscope, or by one of the abovementioned interventions is often not evident, as most of them were commonly reported as ‘fetal surgery’. Our objective is to review and discuss experimental and clinical experience of fetal minimally invasive bimanual surgery and pointing out possible directions for the minimally invasive fetal surgical treatment of congenital malformations.

## 2. Materials and Methods

### 2.1. Literature Research Strategy and Study Selection

**Figure 1:**
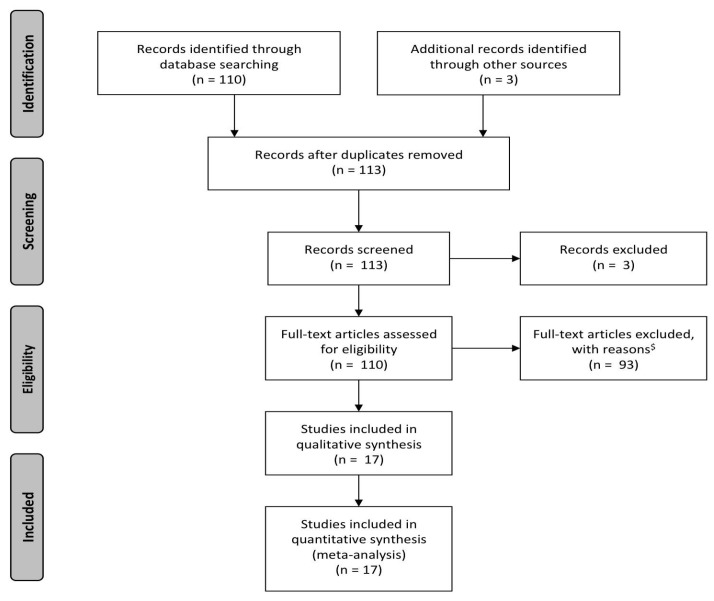
The PRISMA flow chart of evaluation and critical inclusion of the reviewed reports. ^$^: not reporting on minimally invasive bimanual fetal surgery.

The literature search was performed according to the methods previously reported (Figure 1) [5].

### 2.2. Quality Assessment and Data Extraction and Statistical Analysis

For quality assessment, data extraction, and statistical analysis, we used the same methods that have been reported previously [5].

## 3. Results

A total of 17 reports were identified, including 4 animal and 13 human studies. The techniques, methodology and limitations regarding the procedures are reported according to the malformation or indication for fetal surgery. The extracted data was either too heterogenous or the groups too small to perform any statistical analysis. We therefore decided for a narrative reporting of our results and abstained from displaying a large table with non-statistical data to decrease redundancy.

### 3.1. Cardiac Defects

Prenatal interventions for congenital heart defects, such as aortic valve obstruction, date back to 1989 [6]. Since then, two centers have established fetal interventions for intracardiac procedures, namely Boston, USA and Linz, Austria [7]. As the procedures can be performed by direct puncture, depending on the position of the fetus, the Boston group performs a maternal mini laparotomy to improve fetal positioning [7,8].

Kohl et al. reported a case of fetoscopically assisted fetal posturing for balloon valvuloplasty for severe semilunar valve obstruction, as the fetus presented with an unfavorable position. Three 11 Fr catheter sheaths were percutaneously placed into the amniotic cavity, which was then insufflated with carbon dioxide (PACI); the fetus was maneuvered by two graspers to make the following intervention possible [9].

### 3.2. Lower Urinary Tract Obstruction (LUTO)

Congenital malformations leading to urinary tract obstruction result in oligohydramnios, pulmonary hypoplasia, renal and fetal loss. Fetal decompression of the bladder or urinary tract may restore amniotic fluid and salvage both renal and pulmonary function, ultimately to save the child.

The overall indication of fetal surgery for LUTO as well as the optimal technique has yet to be determined, as transabdominal catheter shunt decompression is often inadequate and does not allow for cycling of the bladder, while open procedures cause significant maternal morbidity [10].

Estes et al. reported a fetoscopic two-port approach for vesicoamniotic stenting with an expandable wire mesh wall stent in two sheep fetuses who underwent open fetal surgery for urethral ligation beforehand in order to produce a lower urinary tract obstruction. There was no reported morbidity or mortality related to this procedure, the stent was patent without evidence of peritoneal urine leakage and the urinary system appeared less dilated than the one of the untreated control-fetus [11].

All other reports on animal models or human application of vesicoamniotic shunting or fetal cystoscopic interventions were also not performed bimanually with three ports except for the approach by Quintero et al. who reported a fetal cystoscopy-assisted insertion of vesicoamniotic shunts in four human cases [12].

### 3.3. Congenital Diaphragmatic Hernia (CDH)

Fetal surgery for the correction of congenital diaphragmatic hernia dates back to the beginning of Michael Harrison’s surgically open approach to affected fetuses in the 1980s [13].

In 1997, a three-port fetoscopic approach was applied for tracheal occlusion with an endoscopic clip in one human case at 29 weeks of gestational age: a 2-mm trocar was inserted into the amniotic cavity under ultrasound guidance and a 14 G fetoscope was placed through the port to directly visualize the amniotic placement of an additional 2-mm, 4-mm, and 10-mm port. In order to allow for a bimanual dissection of the neck with scissors the fetoscope was then switched to a 4-mm instrument for better visualization. During this manipulation of tissues, the trachea could be identified and successfully clipped. The fetus survived the procedure and was delivered by EXIT (ex utero intrapartum treatment) at 33 weeks of gestational age, through which the clips could be removed without complication. Due to suffering from Pterygian syndrome and subsequently withdrawing postnatal support, the baby died on the second day of life [14].

Harrison et al. reported on eight fetuses treated by the same technique in 1998 by a four-port approach: they applied stay sutures through the fetus’ chin to fasten it to the uterine wall for better exposure of the neck and put T-fasteners through the trachea for its midline identification. The postnatal outcome of the fetoscopically treated fetuses appeared to be superior to open fetal surgery or postnatal care. Concerning complications of fetoscopic surgery, partial chorioamniotic separation developed in six patients and PPROM (preterm premature rupture of membranes) occurred in five of the eight fetoscopically treated cases [15].

A modification of the fetoscopic technique was reported by Skarsgard in four fetal sheep: instead of clipping the trachea from the outside after surgical dissection of the neck, a water-impermeable polymeric foam plug was inserted into the trachea under fetoscopic visualization and manipulation by an endotracheal route. The number of ports could be reduced from a four to a two-port approach. Although two of the experimental fetuses died after the procedure on postoperative days 1 and 26, the examination performed post-mortally revealed internal lung growth, with intact endotracheal occlusion [16].

In a randomized human trial of fetal tracheal occlusion for severe CDH, the first two fetuses underwent a three-port fetal endoscopic tracheal clip procedure as described above, which was then changed to a single-port balloon-occlusion technique in the following nine fetuses. The trial had to be put to an early end because of the unexpectedly high survival rate with standard postnatal care [17].

Further refinement of this multi-port intraluminal tracheal occlusion technique resulted in a single-port approach with an operative fetoscope and the TOTAL-trial, which demonstrated an improved survival of selected fetuses after tracheal occlusion. However, as no multiport bimanual surgery was performed, those reports had to be excluded [18,19].

### 3.4. Congenital High Airway Obstruction (CHAOS)

Kohl et al. reported a case of a fetus who was suffering from Fraser syndrome as well as CHAOS and ended up at 19 + 5 weeks of gestational age at the time of surgery. The fetus was approached by using three ports, measuring 5 mm each in outer diameter, which were inserted via Seldinger technique to consequently make use of amniotic carbon dioxide insufflation to allow for fetal manipulation and posturing. Utilizing ultrasonographic guidance, the trachea could be decompressed by the means of fetal laryngoscopy with a 2-mm-0◦ rod-lens fetoscope under maternofetal general anesthesia. Roughly nine weeks later the baby had to be delivered via EXIT, mainly because of PPROM [20].

Nicolas et al. reported the fetoscopically assisted needle puncture of the enlarged distal tracheal segment in laryngeal atresia in one human case at 27 weeks of gestational age. The child was delivered three weeks later via EXIT due to PPROM and survived with the assistance of tracheostomy [21].

### 3.5. Open Neural Tube Defects (ONTD)

Fetal surgical repair of ONTD has been associated with improved early neurological outcome compared to the postnatal operation. The treatment consists of the closure of the myelomeningocele and therefore prevention of ventriculomegaly and its complications as well as other neurologic, urologic, musculoskeletal and dermatologic comorbidities throughout the patient’s life [22].

The fetoscopic minimally invasive approach was initially reported by Bruner et al. at Vanderbilt University in 1999 [23] and by Farmer et al. at the University of California, San Francisco, in 2003 [24]. Both techniques required maternal laparotomy by placing three radially expanding ports through the uterine wall in order to bimanually complete a two-layer fetoscopic repair of the defect. Results were disappointing, with a high perinatal mortality, and no further research into fetoscopic approaches occurred for almost two decades in the USA. Instead, an open hysterotomy approach was developed and studied at the centers of the MOMS trial [25].

In 2006, however, Thomas Kohl developed a completely percutaneous fetoscopic technique with an extensive dissection and patch repair [26,27,28] using laparoscopic instruments with both hands. It involves maternal transabdominal ultrasound-guided placement of three to five percutaneous intrauterine 5-mm ports, partial evacuation of amniotic fluid and carbon dioxide insufflation (PACI).

Almost simultaneously, in São Paulo, Brasil, Lapa (Pedreira) et al. developed a similar technique of percutaneous fetoscopic patch repair. Surgery was performed bimanually using three 4- to 5-mm ports and PACI [29]. The neural placode was released using a circumferential incision at the transition zone and the skin was undermined to allow approximation of the edges in the midline. The placode was covered with a biocellulose patch and, if primary skin approximation was feasible, the skin was closed over the patch with a non-absorbable polypropylene running suture. Skin defects that were too great to cover by primarily approximating the edges of the skin were instead closed by using two different patches, consequently positioning a bilaminar skin substitute on top of a biocellulose patch [30].

Results from these two groups have progressively been improving but detractors point out that compared with MOMS data, fetoscopic repair is still associated with a higher rate of prematurity and nearly a doubled incidence of PPROM and a higher perinatal mortality rate and less than equivalent neurological outcomes [31].

Belfort et al. described a fetoscopic approach that mitigates the complications of PPROM in percutaneous fetoscopic repair [32]. These reports have been excluded as a maternal laparotomy does not count as being minimally invasive, but due to the significant reduction in PPROM with their technique, their work is worth mentioning. Distinct from Kohl or Lapa (Pedreira), they accessed the uterus through a maternal laparotomy and completed the operation using PACI by inserting only two 4-mm uterine ports after plicating the membranes against the uterine wall with sutures. A modified, straight endoscope allowing for a passage of a 1.6-mm grasper in combination with fetoscopic instruments were used through these ports [33]. They dissected the neural placode and primarily closed both the dura and the skin as a single layer over the spinal cord, which they did not cover with a patch. After completion the uterine port sites were closed with absorbable sutures. Their approach resulted in 100% fetal and neonatal survival and only 36% babies were delivered at less than 37 weeks of gestational age, which is lower compared to any previously reported cohort of fetoscopic ONTD repair. Theirs was also the first reported cohort study that demonstrated the possibility of vaginal delivery after fetal ONTD repair [18,31,32,33].

### 3.6. Gastroschisis

Kohl and Kahl reported the evaluation of a minimally invasive fetoscopic treatment for gastroschisis in a fetal sheep model [34,35]. Those reports were excluded from our review because maternal laparotomy and hysterotomy were performed, the fetus was exteriorized, placed onto the maternal abdomen and manipulated with the fetoscopic instrumentation. Again, assessing fetoscopic options through a maternal laparotomy and hysterotomy does not represent a minimally invasive fetal procedure.

Our own group reported a similar fetoscopic approach for the creation of gastroschisis in fetal sheep. In contrast to Kohl and Kahl, we were able to perform a second completely percutaneous fetoscopy between 7 and 28 days after the creation of the gastroschisis, thus allowing the herniated intestinal loops to be exposed to the amniotic fluid in order to mimic its pathogenesis as closely as possible. Chorioamniotic separation occurred frequently and hampered with the fetoscopic instrumentation, especially due to us not using an air or carbon dioxide insufflation technique but instead performing amniodistension by amnioinfusion of a physiological saline solution and us consequently working in an under-water environment [36,37,38,39]. Fetoscopic coverage of the intestine was performed with sterilized condoms and a locking running suture (V-Loc™). Postnatal evaluation of the intestine demonstrated a decrease in intestinal damage and re-convalescence of reduction in ICC cells in treated fetuses compared to untreated gastroschisis.

No reports were found of fetoscopic surgery for gastroschisis in humans. Kohl reported fetoscopic visualization of a fetus with gastroschisis, but a surgical intervention was not performed [34].

### 3.7. Miscellaneous Interventions

In 1994, Quintero et al. reported the fetoscopic ligation of the umbilical cord of an anencephalic and acardiac twin at 19 weeks of gestational age. Access to the umbilical cord was gained by a two-port percutaneous approach, with an operative fetoscope and 5 Fr semi-rigid instruments in each 12-gauge port [40].

One year prior to the abovementioned venture, McCurdy reported a similar fetoscopic technique (three-port approach, 5-mm port and endoscope, two 3-mm ports for the instruments and surgical extracorporeal slinging knot) but the fetus died during the procedure [41].

Fetoscopic bimanual repair of cleft lips has been studied in fetal sheep models, but Estes described an open approach to the uterus, which cannot be regarded as a complete minimally invasive procedure [42].

## 4. Discussion

### 4.1. Defining Minimally Invasive Bimanual Fetal Surgery

The term ‘fetal surgery’ is interpreted differently throughout the published reports on fetal treatments. It includes a vast variety of technical applications such as laser ablation by inserting a laser fiber into the fetus, ultrasound-guided interventions with needles, open fetal surgery and fetoscopic single-port interventions [1,2,3,4]. This results in a wide range of techniques, even for the same prenatal intervention, labeling everything as ’fetal surgery’. As surgeons, we define surgery as a medical intervention which is performed with both hands of the surgeon and is visually controlled by either their direct or by endoscopic vision. In contrast to fetal interventions by needles or operative fetoscopes, bimanual surgery may offer improved dexterity, which is needed for the complex repair of selected congenital malformations. To differentiate from one handed ultrasound-guided or fetoscopic single-port interventions, we would like to introduce the somewhat unwieldy but concise term ‘bimanual minimally invasive fetoscopic surgery’.

When reducing the term fetal surgery to the definition of bimanual manipulation of the fetus and focusing on minimally invasive procedures, the field becomes much smaller. The bimanual aspect of the currently reported fetoscopic procedures often relates to fetal posturing for the interventional procedure which is most often the insertion of a needle or a laser fiber.

### 4.2. Minimally Invasive Bimanual Fetal Surgery of Specific Malformations

#### 4.2.1. Cardiac Defects

For cardiac defects, no minimally invasive bimanual procedures were reported, except of fetoscopically assisted fetal posturing for balloon valvuloplasty, which was itself performed by a catheter. Minimally invasive approaches have not been seriously pursued any further, possibly due to an unfavorable risk-benefit ratio and satisfactory prenatal treatment options, such as administering dexamethasone to mothers with Sjögren-syndrome whose fetuses display early signs of complete heart block [43], or sufficient postnatal treatment options for a variety of congenital heart defects. Furthermore, surgical access to the cardiac defect in a beating heart is cumbersome in newborns and therefore much more complex when tried prenatally [7].

#### 4.2.2. Lower Urinary Tract Obstruction (LUTO)

The main intervention for lower urinary tract obstruction (LUTO) is the ultrasound-guided insertion of a draining catheter either into the bladder or into the renal pelvis. Bimanual interventions are rare and reports only give an account of fetoscopically assisted catheter insertions. This may be related to the most common origin of LUTO being in the urethra, hence deep inside the pelvic floor, which even in open postnatal surgery is hard to access surgically. Therefore, endoluminal endoscopic techniques appear to be better suited for LUTO than surgery itself [44].

#### 4.2.3. Congenital Diaphragmatic Hernia (CDH)

A huge effort on surgical research was made for the prenatal treatment of congenital diaphragmatic hernia (CDH). From open surgery in animal models and first human cases, the instruments and technique evolved to a single-port approach to the fetal trachea with an operative fetoscope for temporary balloon occlusion [18,19]. Three- or four-port procedures have been reported in animals and humans during evolution of the tracheal occlusion technique but are not actually needed for simple tracheal occlusion [17]. As tracheal occlusion is merely a palliative or symptomatic, but not a causal management of CDH, the surgical correction of the defect and abdominal enlargement for the intestine with a silastic patch (abdominoplasty) has been evaluated by open fetal surgery in animal studies and human cases [45]. Be that as it may, a report on human interventions in 1993 revealed a high rate of complications and only four surviving fetuses out of fourteen operated on by open surgery [46], concluding that the postnatal outcome of fetoscopically treated fetuses appeared to be superior to open fetal surgery or postnatal care. In addition, it is worth mentioning that these mothers and fetuses who underwent the fetoscopic intervention received dexamethasone beforehand, a steroid that was not antenatally given to the group of children who were only receiving postnatal care, perhaps contributing to the good lung function in the intervention-group after birth [15]. A minimally invasive approach to the primary repair of CDH has not been reported yet, leaving fetoscopic tracheal occlusion as the current state of the art for the prenatal management of CDH. Whether a minimally invasive fetoscopic repair or abdominoplasty for CDH is superior to tracheal occlusion has yet to be evaluated in animal models.

#### 4.2.4. Congenital High Airway Obstruction (CHAOS)

Fetal surgery for congenital high airway obstruction (CHAOS) consists of tracheal puncture for decompression. The intervention can also be performed in the fetal larynx by a single fetoscope. In selected cases bimanual posturing and manipulation of the fetal position for an optimal angle to allow secure entering of the decompression needle was essential for the success of this procedure. A true three-port approach and bimanual intervention for CHAOS has not been reported so far. Moreover, in CHAOS the direct surgical approach to the sublaryngeal region of the obstruction is challenging and a procedure difficult to perform even in newborns under EXIT [47].

#### 4.2.5. Open Neural Tube Defects (ONTD)

Open neural tube defects (ONTDs) are frequently addressed by fetal surgery as the defects on the back side of the fetus are easily accessible. The current bimanual techniques in fetoscopic surgery range from two-port single-layer repair after maternal laparotomy to three- or multi-port completely percutaneous procedures that use full thickness or multi-layer closure of the defect with a variation in neurologic and obstetrical outcome as well as perinatal mortality. This illustrates that techniques should be optimized, and that standardization has yet to be achieved. The Belfort technique is not completely minimally invasive as it requires a maternal laparotomy and exteriorization of the uterus. Thus, the search for a truly maternal and fetal minimally invasive endoscopic technique must be pursued and rigorously tested for in experimental and clinical trials.

#### 4.2.6. Gastroschisis

For the prenatal management of gastroschisis, only one minimally invasive technique was reported: the percutaneous fetoscopic covering of the intestine was feasible in a fetal sheep model. As the coverings dislocated from the fetuses during gestation, a refinement of the technique and instruments appears warranted before any further thought of human application can be considered. Although gastroschisis is a congenital malformation which is easily accessible for fetal surgery as spina bifida repair, not many efforts have been made in order to improve reported fetoscopic techniques. This may be due to gastroschisis generally being accounted for as a rather benign congenital malformation with an excellent postnatal outcome. However, recent analyses revealed a distinct and increased morbidity and mortality in cases of complex gastroschisis [48,49]. This insight might lead to a novel fetal surgical approach, especially for the complex cases of gastroschisis.

### 4.3. The Rationale of Minimally Invasive Fetal Surgery

**Figure 2:**
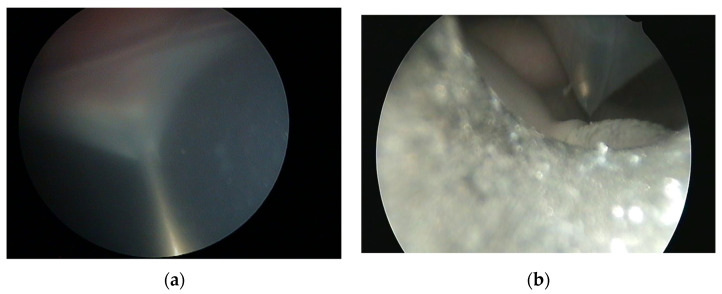
Fetoscopic view of the detachment of uterine membranes in a sheep model during insertion of a 16 G needle for port placement (**a**) and insertion of a 3 mm port (**b**). Below the trocar the umbilical cord can be seen.

In general surgery, the minimally invasive approach to the surgical field promises to offer less pain, faster recovery, smaller scars, less time to full diet and less postoperative complications, although contradicting data can also be found in double blind randomized trials [50,51,52]. The logical conclusion is that the minimally invasive approach to the fetus could offer the same benefits to the mother and to the child as seen in general surgery. However, complicating the fetal surgery, in contrast to classic minimally invasive surgery, is the circumstance that fetal surgery not only affects the fetus itself but also the mother as well as her uterus. Therefore, the uterus has to be seen as a separate and third entity with its own set of complications that may also affect the fetus or the mother. The approach to the fetus by hysterotomy is associated with risks that are inherent to the uterine incision. Preterm premature rupture of membranes (PPROM) is a dreaded complication after uterine disturbance and could be proven to be more frequently encountered after percutaneous compared to open fetal surgery [31]. The following Figure 2 showcases the cause of the abovementioned phenomenon that our group caused by inserting a 16 G needle through the uterus of a sheep.

The prematurely delivered babies therefore suffer not only from their congenital malformation but also from all aspects of prematurity. It appears that the fixation of the chorioamniotic membranes to the uterine wall, as it is performed in open fetal surgery by applying a stapling device for opening the uterus with synchronous stapling of the membranes, prevents their separation (chorioamniotic separation, CAS) and subsequently averts PPROM from occurring. The technique of chorioamniotic plication to the uterine wall before insertion of the ports for fetal surgery (maternal laparotomy, uterine exteriorization and plication, followed by fetoscopy) exploits this technique and leads to a reduction in PPROM in hybrid fetoscopic surgery [32]. Although being the best current option for minimally invasive access to the fetus, concerning the integrity of the mother, the hybrid technique does not count as minimally invasive surgery. Only a minimally invasive approach to all three entities involved should be considered truly ‘minimally invasive’.

Interventional procedures such as ultrasound-guided punctures or laser ablations do not count as surgery per sé. However, we do recognize that the risk of complications is reduced with every averted trauma to the uterus, therefore reducing the number of operative ports to as little as possible still enables some interventions, such as the prenatal repair of spina bifida, to be considered ‘surgery’, as long as both hands of the surgeon are used. It is noticeable that the small operating field usually requires secure posturing of the fetus which often can only be acquired by the usage of both hands of the surgeon.

Last but not least: we deem the new contribution of this review being that we ask for a more clearer definition of the term “fetal surgery” and especially “minimally invasive fetal surgery”: is the FETO procedure minimally invasive? It appears so, as only one small diameter operative fetoscope is used; but is it surgery? Some surgeons may not agree and would rather call it an intervention than a surgical procedure. Is the Belfort technique fetal surgery? Many will agree, as one operates the fetus with instruments in both hands. However, is the Belfort technique really a minimally invasive procedure—as you need to cut the mother open in order to obtain access to her uterus?

We therefore hope to spark a discussion of which procedures should be called “minimally invasive” or fetal “surgery” compared to fetal “interventions” by defining the new term “bimanual minimally invasive fetoscopic surgery”. Whether procedures performed by this approach are truly minimally invasive must then be evaluated, but at least can from now on be compared to each other.

### 4.4. Ethical Considerations of Bimanual Minimally Invasive Fetal Surgery

Although this review primarily discusses technical aspects of bimanual minimally invasive fetal surgery, which does not require an ethical discussion per sé, we still found ourselves caught up in an ethical dilemma.

This approach of fetomaternal surgery first and foremost is a minimally invasive operation performed on the mother, resulting in reduced risks and severity of complications usually associated with open surgery. However, this advantage does not pose a minimally invasive operation to the fetus who will likely face an increased risk of preterm premature rupture of the membranes (PPROM) [53] and possibly suffer from adverse effects of prematurity [54].

Alternatively, to decrease the probability of PPROM occurring, one could perform open fetoscopy according to Belfort [32,53], but would again have to put the mother at risk by performing open surgery on her. Possible solutions could be operating later on in gestation, which might miss the point of prenatal treatment entirely, or continuously decreasing the size of the incision performed on the uterus in a feasible manner, finding ways to close off the insertion side of the trocar sustainably, or humidifying the membranes [55,56,57,58].

Beyond that, we also emphasize the importance of the implementation of an adequate animal model for the individual issue at hand before advancing to human case studies but question the alleged necessity of killing each and every animal, meaning mother and offspring that have been experimented on, at the end of any trial.

Realizing our suggested algorithm of firstly experimenting on inanimate objects, then advancing to deceased animals and subsequently to live animal models, in order to allow for the progression to human cases, would reduce the overall harm to the animals who are experimented on [5]. Logically, an animal mother should either only carry one fetus or, if not compatible with the hypothesized approach or proven impractical, carry more fetuses which consequently are to be utilized; none are to be discarded. If applicable, the animal mother should be allowed to survive beyond the end of a trial; releasing her or using her for future trials would reduce the total number of animals we experiment on and kill.

Furthermore and despite all technical considerations, any fetal surgical intervention should only be applied when the rules for fetal interventions are followed, such as: the malformation should be able to be diagnosed by prenatal ultrasound, it should have a high postnatal morbidity and mortality, a standardized fetal intervention should be available which reduces postnatal morbidity and mortality and has to be proven effective in inanimate an animate models of the disease and last but not least, the fetal intervention does not have to mean harm to neither the mother, her uterus nor the fetus itself.

The purpose of this review is to emphasize the reported differences between fetal interventions and fetal surgery, especially minimally invasive fetal surgery. The line between ultrasound guided needle interventions (not that seldom called fetal surgery by the interventionists themselves), single-port access operative fetoscopic techniques (such as FETO) and multi-port endoscopic fetal surgery (the Belfort-technique for example) is much too often thin and unclear. As a result of this mixture of nomenclature for completely different types of fetal interventions (as demonstrated in the high heterogeneity of the reports included in an excluded from this review), each with its own risk profile and procedure specific complications, we deem a strict definition of the applied techniques of fetal intervention much more than just an academic effort. Only with a strict definition of the techniques used in surgical procedures will comparable studies be possible and result in level 1 evidence on the effect and—hopefully—benefit of “fetal surgery”.

## 5. Conclusions

Most minimally invasive fetal interventions are single-port fetoscopic or ultrasound-guided procedures. Bimanual minimally invasive fetal surgery is rare. For Spina bifida repair in humans, in contrast to the open procedures performed in the MOMS trial [25], several three-port approaches with bimanual fetal surgery have been established. Whether one approach is superior to the other has yet to be evaluated. A completely minimally invasive percutaneous three-port approach to the fetus has been established in an animal model for the management of gastroschisis, but human application is missing.

Summarizing, it can be stated that, except for the treatment of open neural tube defects, bimanual minimally invasive surgery is not yet reliably implemented for the surgical treatment of the fetus.

Finding a solution to CAS/PPROM necessitates further scientific effort, otherwise true minimally invasive fetomaternal surgery cannot be achieved safely. Sufficient closure of the insertion side of the port, for instance via plugging or gluing [56] or a modification to Belfort’s technique, require additional investigation.

## Data Availability

Not applicable.
